# One family cluster of avian influenza A(H7N9) virus infection in Shandong, China

**DOI:** 10.1186/1471-2334-14-98

**Published:** 2014-02-21

**Authors:** Ti Liu, Zhenqiang Bi, Xianjun Wang, Zhong Li, Shujun Ding, Zhenwang Bi, Liansen Wang, Yaowen Pei, Shaoxia Song, Shengyang Zhang, Jianxing Wang, Dapeng Sun, Bo Pang, Lin Sun, Xiaolin Jiang, Jie Lei, Qun Yuan, Zengqiang Kou, Bin Yang, Yuelong Shu, Lei Yang, Xiyan Li, Kaishun Lu, Jun Liu, Tao Zhang, Aiqiang Xu

**Affiliations:** 1 Shandong Provincial Center for Disease Control and Prevention; Shandong Provincial Key Laboratory of Infectious Diseases Control and Prevention, Academy of Preventive Medicine, Shandong University, Jinan 250014, Shandong, China; 2 National Institute for Viral Disease Control and Prevention, Chinese Center for Disease Control and Prevention, Beijing, China; 3 Zaozhuang Center for Disease Control and Prevention, Zaozhuang, Shandong, China

**Keywords:** Avian Influenza A (H7N9) virus, Epidemiology, Infectious source

## Abstract

**Background:**

The first case of human infection with avian influenza A (H7N9) virus was identified in March, 2013 and the new H7N9 virus infected 134 patients and killed 45 people in China as of September 30, 2013. Family clusters with confirmed or suspected the new H7N9 virus infection were previously reported, but the family cluster of H7N9 virus infection in Shandong Province was first reported.

**Case presentation:**

A 36-year-old man was admitted to Zaozhuang City Hospital with progressive respiratory distress and suspicion of impending acute respiratory distress syndrome on April 21. The chest radiography revealed bilateral ground-glass opacities and pulmonary lesions. The second case, the first case’s 4 year old son, was admitted to the same hospital on April 28 with fever and multiple patchy shadows in the bilateral lungs. Both of the two cases were confirmed to infect with H7N9 virus by the results of real-time reverse transcriptase-polymerase-chain reaction (rRT-PCR), virus isolation and serum antibody titer. At the same time, one environment samples was detected positive for H7N9 virus in the living poultry market in Zaozhuang. The homologous analysis of the full genome sequence indicated that both viruses from the patients were almost genetically identical. The field epidemiology investigation showed that the two cases had no recognized exposure to poultry, but had the exposure to the environment. The second case had substantial unprotected close exposure to his ill father and developed symptoms seven days after his last contact with his father. After surgery, the index case and his son were discharged on May 16 and May 6, respectively. 11 close contacts of both patients were identified and tested negative both the throat swabs and the serum antibody.

**Conclusion:**

The infection of the index case probably resulted from contact with environmentally contaminated material. For the son, the probable infection source was from the index case during unprotected exposure, but the possibility from the environment or other sources could not be completely ruled out.

## Background

The first case of human infection with a novel reassortant avian influenza A (H7N9) virus was identified in Shanghai and Anhui of China during February and March 2013 [1-3]. Since this H7N9 virus was not detected in humans or animals previously, the situation raises many urgent questions and global public health concerns. The H7N9 virus infections have extended to 12 provinces infected 134 patients and killed 45 people in China as of September 30, 2013 [4]. In most of the laboratory confirmed cases the patients developed severe pneumonia and acute respiratory distress syndrome (ARDS) and needed intensive care [5].

According to the available epidemiological data, most infected patients had a history of visiting live poultry markets or contact with poultry and the mean incubation period was 3.3 days, indicating that the sources of infection were likely to be either contaminated environment or infected poultry [6-10]. Though limited human to human spread could not be ruled out in three families, no clear evidence indicated that the novel virus could transmit from person to person [11,12].

Shandong’s first human H7N9 case was confirmed on 23^rd^ April, and then the second human H7N9 case who was the son of the index case was confirmed on 28^th^ April. In this report, we report the family cluster with H7N9 virus infection.

## Case presentation

### Patients

**Figure 1:**
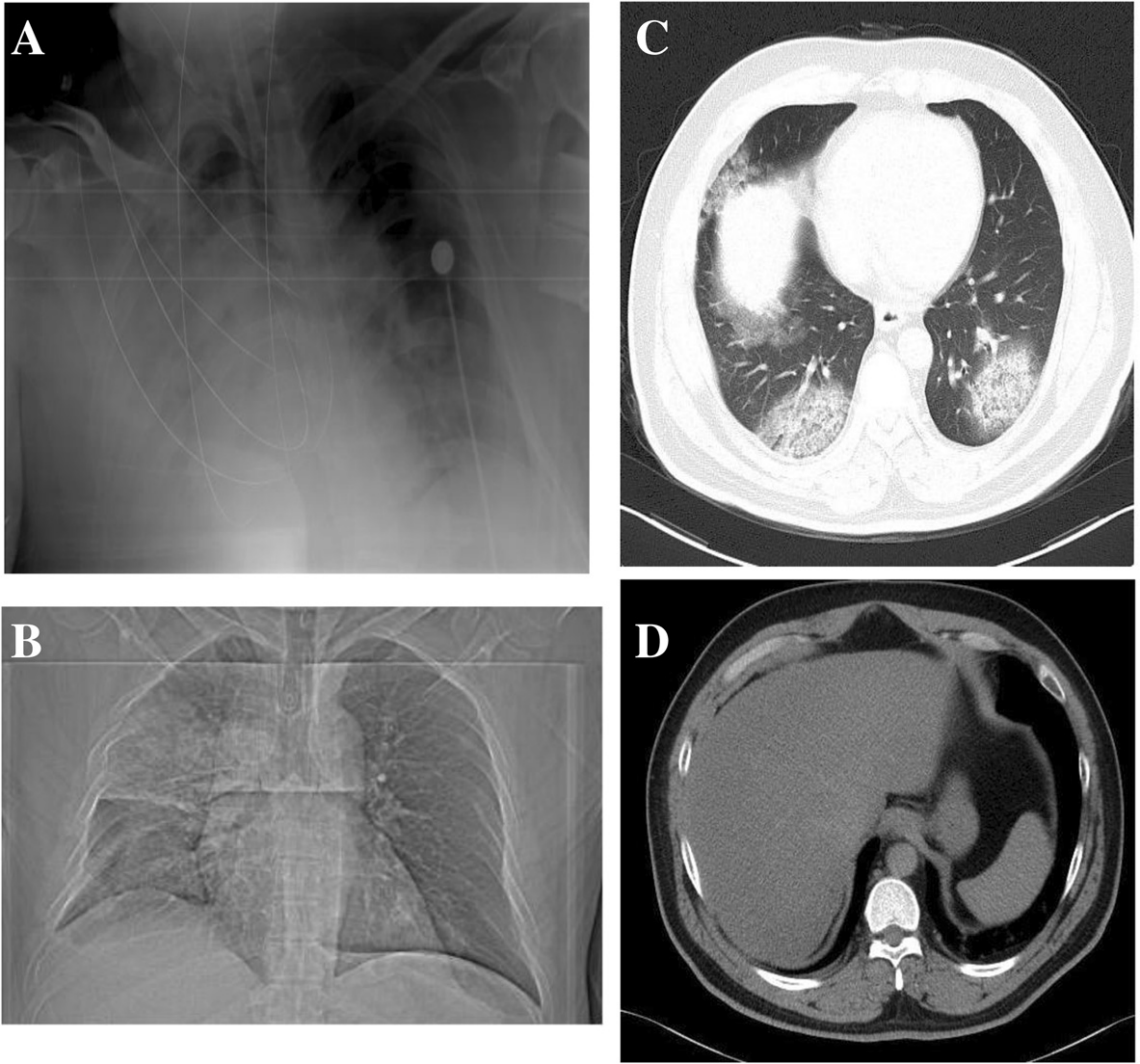
**A chest radiograph of the index case is shown in Panel A and B on day 6 after the onset of the illness.** A computed tomography scan of the chest of the index case is shown in Panel **C** and **D** after the onset of the illness.

The index case, 36 year old man, lived with his wife, two daughters and his son. He presented to a clinic with fever (37.8°C) and cough on April 16. He felt well after taking compound paracetamol tablets (II), Ibuprofen Tablets and Radix Isatidis on April 17. On April 18, he continued fever and developed pneumonia and was admitted to Zaozhuang City Hospital on April 21. Due to progressive respiratory distress and suspicion of impending acute respiratory distress syndrome, he was transferred to intensive care unit on the day of admission. Bilateral ground-glass opacities and pulmonary lesions were observed on chest radiography (Figure 1). The index patient recovered and was discharged on May 16. The positive throat-swab collected on April 21 was confirmed by Chinese Center for disease Control and Prevention on April 23 and virus was A/Shandong/01/2013(H7N9) (SD/01). The antibody titers was 1:80 for the serum sample collected on May 21^st^ compared with 1:5 on April 24 by the micro-neutralization assay [13].

The second case was the index patient’s 4 years old son. He began to have fever (37.8°C) and cough on April 14. He was given domiciliary care before April 18. From April 18 to 20, he was treated for common cold at the clinic with his father and recovered. On 21^st^ April, when the index case was hospitalized, the boy separated with him and was under medical observation till April 27 when the fever (38.3°C) and cough started again. After taking antipyretic granules and cephalosporin on the morning, the temperature decreased to 37°C in the afternoon. On 28th morning, he was hospitalized with fever (37.2°C) and was treated with oseltamivir (5 mg). Initial testing identified leucopenia and multiple patchy shadows in the lungs bilaterally. He was discharged on May 6. The throat swab collected on April 23 was negative for H7N9. On April 27, PCR test from throat swabs was H7N9 positive and virus was A/Shandong/0068A/2013(H7N9) (SD/0068A). The antibody titers was 1:20 for the serum sample collected on May 21^st^ compared with 1:5 on April 24 and May 1^st^.

Table 1 summarized the demographic and clinical characteristics of the two cases.

**Table 1:** Demographic, epidemiology, and Virologic characteristics and complications, treatment and clinical outcomes of two patients infected with H7N9 virus

**Characteristic**	**Patient 1**	**Patient 2**
Age (yr)	36	4
Sex	Male	Male
Occupation	Individual practitioners	Kindergarden
Underlying conditions		
Chronic smoker	Yes	No
Exposure to living animal in past 7 days	Yes	Yes
Date of illness onset	April 16, 2013	April 27, 2013
Date of admission	April 21, 2013	April 28, 2013
Admission to ICU	April 21, 2013	No
Presenting symptoms		
Temperature (°C)	38.8°C	38.3°C
Sore throat	+	-
Rhinorrhoea	-	-
Conjunctivitis	-	-
Cough	+	+
Sputum	+	+
Haemoptysis	-	-
Dyspnoea	+	-
Nausea or vomiting	-	-
Diarrhoea	-	-
Abdominal pain	-	+
Myalgia	-	-
Fatigue	+	-
Skin rash	-	-
Findings on admission		
Respiratory rate(breaths/min)	33/min	20/min
Total White-Cell count(per L)	3.3*10∧9	2.0*10∧9
Absolute lymphocyte count(per ml)	0.7*10∧7	6.4*10∧9
Platelet count(per L)	133*10∧9	394*10∧9
Time between onset of symptoms and initiation of oseltamivir(days)	6 days	1 days
Time between onset of symptoms and onset of respiratory failure (days)	6 days	No
Time between onset of respiratory failure and need for mechanical ventilation (hours)	4 h	No

### Epidemiological investigation

**Figure 2:**
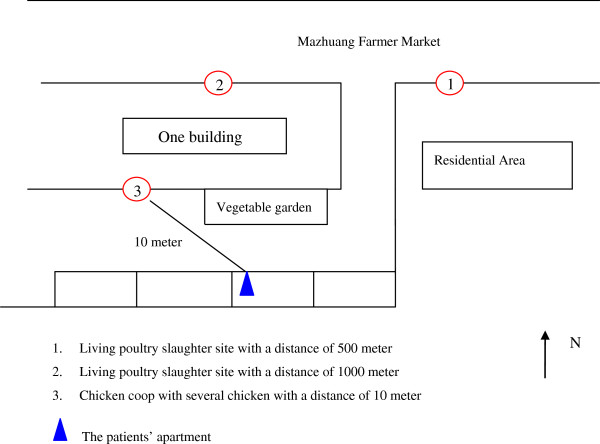
The surround environment of the confirmed H7N9 case patients.

**Figure 3:**
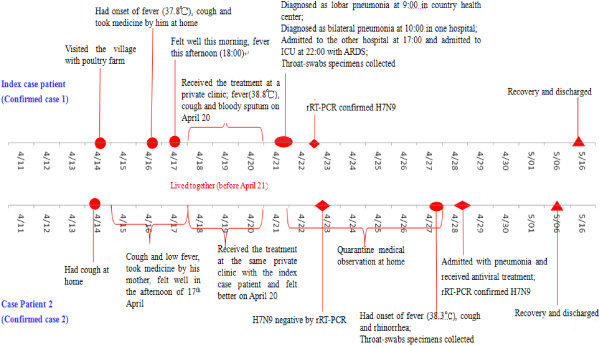
**Timeline of patient exposures and dates of illness onset in the family cluster of cases of H7N9 Virus infection.** Note: ICU denotes intensive care unit, and rRT-PCR real-time reverse-transcriptase–polymerase-chain-reaction assay.

The index case lived in rural–urban fringe zone in Zaozhuang City. There were several living chicken in the cage and vegetable gardens fertilized with fences in the living community away 10 meters from their apartment. And there were two living poultry slaughter sites in the Mazhuang Farm Market located 500 and 1000 meters from the residential district (Figure 2).Interviews with the family members, other close contacts and the first case after recovery constructed the timeline of the two cases onset of ill and treatment (Figure 3). From April 6 to 16, the index case always stayed at home except taking his two daughters to school on the morning and picking them up after school every day by car with his wife and his son, while they passed the poultry slaughter sites in the Mazhuang Farm Market. Two days before the onset of illness, the index case had visited a village with several large poultry farms in the other city, but he didn’t enter the poultry farms. At the same time, his son didn’t go to the kindergarten and stayed at home since April because he always got sick and had a cold on April 14.

Furthermore, the index case was not in charge of purchase the foodstuff and his family members didn’t buy poultry from the two living slaughter sites in the Mazhuang Farm market and the other markets. None of the members of his family raised poultry or other animals, none brought live poultry to their home, none had direct contact with sick or dead poultry, and none played with the living chicken in the living community.

After the index case fell ill, his family, including his son had prolonged, close, unprotected contact with him, including eating together. Furthermore his son was the nearest to him when they ate, watched TV and slept till April 20. His son also accompanied the index case while seeking medical care from April 18 to 20. After the index case hospitalization, his son was under the medical observation till April 27 in his apartment and didn’t go out.

11 close contacts of the two H7N9 cases were identified and followed up daily for 7 days. Of these, seven were family members or relatives, two were health care workers and two were index case’s friends. All the throat swabs collected on April 23 were negative for H7N9 virus. Four close contacts of the second case were the same as those of index case, all being family members (the index case’s wife, his two daughters and mother in law), whose specimens collected on April 28 were negative for H7N9. No micro-neutralization antibodies against A (H7N9) virus were detected from paired serum samples of all close contacts. To trance the infectious source, 96 environment samples were collected and only one chopping block swab from Jinniu living poultry market located 10 kilometers away from the living community was positive for H7N9 by rRT-PCR and virus was A/environment/Shandong/01/2013(H7N9) (Ev/SD/01),while the samples from the surrounding areas of the living community including the slaughter sites were negative.

Complete genomic sequencing of SD/01, SD/0068A and Ev/SD/01 were analyzed. The homologous analysis showed that they were 97.92% to 100% identical in all eight gene segments and the two human A (H7N9) viruses were 100% identical in HA, PB1, PA, NS and M genes (Table 2). There are only four amino acid substitutions between the two human A (H7N9) viruses, two in the PB2 (E341K and R427Q substitutions), one in NA (L399S substitution) and one in NP (M191V substitution), respectively. The full-length gene sequence number in GISAID are EPI 1447715–1447721 and 1447618 [SD/01], EPI 1457428–1457435 [SD/0068A], EPI1447645-651 and 1447628 [Ev/SD/01].

**Table 2:** Genome sequence similarity among the three H7N9 virus isolates

**Gene**	**SD/01**	
	**SD/0068A**	**EV/SD/01**
HA	100.00%	99.82%
NA	99.86%	99.93%
PB2	99.91%	99.87%
PB1	100.00%	99.65%
PA	100.00%	97.92%
NP	99.86%	98.76%
M	100.00%	99.79%
NS	100.00%	99.76%

Note: SD/1: A/Shandong/1/2013(H7N9).

SD/0068A: A/Shandong/0068A/2013(H7N9).

EV/SD/1: A/Environment/Shandong/01/2013(H7N9).

## Conclusions

In the H7N9 family cluster, for the index case, we were not able to determine the source of H7N9 virus infection, and transmission through contact with environmentally contaminated material remains a possibility [14]. First, the chopping block swab and the environment samples with H7N9 virus [15,16] suggested the possibility of environmental contamination with H7N9 virus, although no virus was detected around the residence. At the same time, the poultry market has been identified as the infectious source and the risk factor for human infections [8,9]. Second, He always passed the slaughter sites in the Mazhuang Farm Market by car and his car was parked around the chicken cage and vegetable garden, even sometimes was contaminated by the bird feces or chicken fences. Last, the index case had no exposure to poultry or history of visiting live poultry markets and also didn’t have contact with the febrile person prior to the onset.

For the second case, his initial fever date was 14^th^ April, but it was unlikely this was onset date. All the laboratory results were negative and he recovered after common cold treatment. His second time fever date, 27^th^ April, was the onset date. He got infection from two possible routes. The first possible route was he got infection from the index case like other reports published [12,14,17,18]. Firstly, the boy had intensive and close contact with the index case even after index case onset. From April 16, the index case still provided care to his son and they went to the clinic together. And from April 18 to 20, the index case began to expectorate and the illness progressed rapidly. Apart from the depressed immunity because of common cold, the son was nearest to him while having dinner, watching TV and sleeping in the same room. Secondly, except from the living chicken in the community, the son had no exposure to other poultry and rarely walked beyond the apartment before April 21. After the index case hospitalization, the son didn’t go out till April 27. Last, the sequence analysis showed that both virus from patients possessed high degrees of similarity between nucleotide (99.8%-100.0%) and amino acid (99.7%-100.0%) sequence.

The second possible route was the boy had the same exposure as his father sometime before 16^th^ April, then for him the incubation date was more than 11 days, suggesting simultaneous acquisition from a common source was unlikely, because the average incubation period was in three or six days (range, 1 to 10) [11,19]. But the exposure to environmentally contaminated material [14] as his father was a possibility when he went to the clinic with his father from April 18 to 20.

In conclusion, we analyzed the epidemiology characteristics of the family cluster of H7N9 virus, especially the infection source of the two cases. Though it is difficult to ascertain the infectious source for the two cases, but the emergence of H7N9 clusters requires urgent attention because of the possibility that a change in the epidemiological character could spread more easily among people.

## Consent

Written informed consent was obtained from the patient and the guardian for publication of this Case report and any accompanying images. A copy of the written consent is available for review by the Series Editor of this journal. The study was also approved by the institutional review board of Shandong Provincial Center for Disease Control and Prevention.

## Competing interests

The authors declare that they have no competing interests.

## Authors’ contributions

ZB, AX and YS designed the study, XW, SD, ZB, LW, SZ, JL, QY, ZK, BY, KL and JL had roles in recruitment, field investigation and data collection. TL, ZL, YP, JW, BP, TZ, LY and XL had roles in laboratory testing and analysis. DS and XJ prepared the figures. TL, XW, AX and ZB wrote the paper and all authors contributed to review and revision and have seen and approved the final version.

## Pre-publication history

The pre-publication history for this paper can be accessed here:

http://www.biomedcentral.com/1471-2334/14/98/prepub

## References

[B1] World Health Organization Background and summary of human infection with influenza A (H7N9) virus 2013 [http://www.who.int/influenza/human_animal_interface/update_20130405/en/]

[B2] National Health and Family Planning Commission Three confirmed cases of human infection with avian influenza A(H7N9) virus in Shanghai and Anhui 2013 [http://www.moh.gov.cn/mohwsyjbgs/s3578/201303/44f25bd6bed14cf082512d8b6258fb3d.shtml]

[B3] Gao R Cao B Hu Y Feng Z Wang D Hu W Chen J Jie Z Qiu H Xu K Xu X Lu H Zhu W Gao Z Xiang N Shen Y He Z Gu Y Zhang Z Yang Y Zhao X Zhou L Li X Zou S Zhang Y Li X Yang L Guo J Dong J Li Q Human infection with a novel avian-origin influenza A (H7N9) virus N Engl J Med 2013 14 1888 1897 10.1056/NEJMoa1304459 23577628

[B4] National Health and Family Planning Commission Human infections with avian influenza A (H7N9) virus in September, 2013 2013 [http://www.nhfpc.gov.cn/yjb/s3578/201310/1ca7a35a2be54b3fa2c24ef45dade1b5.shtml]

[B5] Gao HN Lu HZ Cao B Du B Shang H Gan JH Lu SH Yang YD Fang Q Shen YZ Xi XM Gu Q Zhou XM Qu HP Yan Z Li FM Zhao W Gao ZC Wang GF Ruan LX Wang WH Ye J Cao HF Li XW Zhang WH Fang XC He J Liang WF Xie J Zeng M Clinical findings in 111 cases of influenza A (H7N9) virus infection N Engl J Med 2013 14 2277 2285 10.1056/NEJMoa1305584 23697469

[B6] Shi J Deng G Liu P Zhou J Guan L Li W Li XY Guo J Wang GJ Fan J Wang JL Li YY Jiang YP Liu LL Tian GB Li CJ Chen HL Isolation and characterization of H7N9 viruses from live poultry markets—implication of the source of current H7N9 infection in humans Chin Sci Bull 2013 14 1857 1863 10.1007/s11434-013-5873-4

[B7] Chen Y Liang W Yang S Wu N Gao H Sheng J Yao H Wo J Fang Q Cui D Li Y Yao X Zhang Y Wu H Zheng S Diao H Xia S Zhang Y Chan KH Tsoi HW Teng JL Song W Wang P Lau SY Zheng M Chan JF To KK Chen H Li L Yuen KY Human infections with the emerging avian influenza A H7N9 virus from wet market poultry: clinical analysis and characterisation of viral genome Lancet 2013 14 1916 1925 10.1016/S0140-6736(13)60903-4 23623390 PMC7134567

[B8] Bao CJ Cui LB Zhou MH Hong L Gao GF Wang H Live-animal markets and influenza A (H7N9) virus infection N Engl J Med 2013 14 2337 2339 10.1056/NEJMc1306100 23697471

[B9] Li J Yu X Pu X Xie L Sun Y Xiao H Wang F Din H Wu Y Liu D Zhao G Liu J Pan J Environmental connections of novel avian-origin H7N9 influenza virus infection and virus adaptation to the human Sci China Life Sci 2013 14 485 492 10.1007/s11427-013-4491-3 23657795

[B10] Hongjie Y Wu JT Cowling BJ Qiaohong L Fang VJ Sheng Z Peng W Hang Z Lau EHY Danhuai G Ni MY Zhibin P Luzhao F Hui J Huiming L Qun L Zijian F Yu W Weizhong Y Leung GM Effect of closure of live poultry markets on poultry-to-person transmission of avian influenza A H7N9 virus: an ecological study Lancet 2013 doi:10.1016/S0140-6736(13)61904-2 10.1016/S0140-6736(13)61904-2 PMC3946250 24183056

[B11] Li Q Zhou L Zhou M Chen Z Li F Wu H Xiang N Chen E Tang F Wang D Meng L Hong Z Tu W Cao Y Li L Ding F Liu B Wang M Xie R Gao R Li X Bai T Zou S He J Hu J Xu Y Chai C Wang S Gao Y Jin L Preliminary report: epidemiology of the avian influenza A (H7N9) outbreak in China N Engl J Med 2013 doi:10.1056/NEJMoa1304617

[B12] Qi X Qian YH Bao CJ Guo XL Cui LB Tang FY Ji H Huang Y Cai PQ Lu B Xu K Shi C Zhu FC Zhou MH Wang H Probable person to person transmission of novel avian influenza A (H7N9) virus in Eastern China, 2013: epidemiological investigation BMJ 2013 14 f4752 10.1136/bmj.f4752 23920350 PMC3805478

[B13] Tian B Jianfang Z Yuelong S Serologic study for Influenza A (H7N9) among high-risk groups in China N Engl J Med 2013 doi:10.1056/NEJMc1305865 10.1056/NEJMc1305865 23718151

[B14] Kandun IN Wibisono H Sedyaningsih ER Yusharmen Hadisoedarsuno W Purba W Santoso H Septiawati C Tresnaningsih E Heriyanto B Yuwono D Harun S Soeroso S Giriputra S Blair PJ Jeremijenko A Kosasih H Putnam SD Samaan G Silitonga M Chan KH Poon LL Lim W Klimov A Lindstrom S Guan Y Donis R Katz J Cox N Peiris M Three Indonesian clusters of H5N1 virus infection in 2005 N Engl J Med 2006 14 2186 2194 10.1056/NEJMoa060930 17124016

[B15] Three samples were detected for H7N9 virus in Zaozhuang City which came from the same market 2013 [http://www.sd.xinhuanet.com/news/2013-05/06/c_115644619.htm]

[B16] Zhang Q Shi J Deng G Guo J Zeng X He X Kong H Gu C Li X Liu J Wang G Chen Y Liu L Liang L Li Y Fan J Wang J Li W Guan L Li Q Yang H Chen P Jiang L Guan Y Xin X Jiang Y Tian G Wang X Qiao C Li C H7N9 influenza viruses are transmissible in Ferrets by respiratory droplet Science 2013 14 6144 410 414 doi: 10.1126/science.1240532 10.1126/science.1240532 23868922

[B17] Wang H Feng Z Shu Y Yu H Zhou L Zu R Huai Y Dong J Bao C Wen L Wang H Yang P Zhao W Dong L Zhou M Liao Q Yang H Wang M Lu X Shi Z Wang W Gu L Zhu F Li Q Yin W Yang W Li D Uyeki TM Wang Y Probable limited person-to-person transmission of highly pathogenic avian influenza A (H5N1) virus in China Lancet 2008 14 1427 1434 10.1016/S0140-6736(08)60493-6 18400288

[B18] Ungchusak K Auewarakul P Dowell SF Kitphati R Auwanit W Puthavathana P Uiprasertkul M Boonnak K Pittayawonganon C Cox NJ Zaki SR Thawatsupha P Chittaganpitch M Khontong R Simmerman JM Chunsutthiwat S Probable person-to-person transmission of avian influenza A (H5N1) N Engl J Med 2005 14 333 340 10.1056/NEJMoa044021 15668219

[B19] Cowling BJ Jin L Lau EH Liao Q Wu P Jiang H Tsang TK Zheng J Fang VJ Chang Z Ni MY Zhang Q Ip DK Yu J Li Y Wang L Tu W Meng L Wu JT Luo H Li Q Shu Y Li Z Feng Z Yang W Wang Y Leung GM Yu H Comparative epidemiology of human infections with avian influenza A H7N9 and H5N1 viruses in China: a population-based study of laboratory-confirmed cases Lancet 2013 14 129 137 10.1016/S0140-6736(13)61171-X 23803488 PMC3777567

